# Genetic Basis of Sjögren's Syndrome. How Strong is the Evidence?

**DOI:** 10.1080/17402520600876911

**Published:** 2006

**Authors:** Juan-Manuel Anaya, Angélica María Delgado-Vega, John Castiblanco

**Affiliations:** 1 Cellular Biology and Immunogenetics Unit Corporación para Investigaciones Biológicas Medellín Colombia; 2 Universidad del Rosario Medellín Colombia

## Abstract

Sjögren's syndrome (SS) is a late-onset chronic autoimmune disease (AID) affecting the exocrine glands, mainly the salivary and lachrymal. Genetic studies on twins with primary SS have not been performed, and only a few case reports describing twins have been published. The prevalence of primary SS in siblings has been estimated to be 0.09% while the reported general prevalence of the disease is approximately 0.1%. The observed aggregation of AIDs in families of patients with primary SS is nevertheless supportive for a genetic component in its etiology. In the absence of chromosomal regions identified by linkage studies, research has focused on candidate gene approaches (by biological plausibility) rather than on positional approaches. Ancestral haplotype 8.1 as well as *TNF*, *IL10* and *SSA1* loci have been consistently associated with the disease although they are not specific for SS. In this review, the genetic component of SS is discussed on the basis of three known observations: (a) age at onset and sex-dependent presentation, (b) familial clustering of the disease, and (c) dissection of the genetic component. Since there is no strong evidence for a specific genetic component in SS, a large international and collaborative study would be suitable to assess the genetics of this disorder.


*Clinical & Developmental Immunology*, June–December 2006; 13(2–4): 209–222

Taylor & Francis

Taylor & Francis Group

### Genetic basis of Sjögren's syndrome. How strong is the evidence?

JUAN-MANUEL ANAYA[1,2], ANGÉLICA MARÍA DELGADO-VEGA[1,2], & JOHN CASTIBLANCO[1]

[1]*Cellular Biology and Immunogenetics Unit, Corporación para Investigaciones Biológicas, Medellín, Colombia, and* [2]*Universidad del Rosario, Medellín, Colombia*

**Abstract**

Sjögren's syndrome (SS) is a late-onset chronic autoimmune disease (AID) affecting the exocrine glands, mainly the salivary and lachrymal. Genetic studies on twins with primary SS have not been performed, and only a few case reports describing twins have been published. The prevalence of primary SS in siblings has been estimated to be 0.09% while the reported general prevalence of the disease is approximately 0.1%. The observed aggregation of AIDs in families of patients with primary SS is nevertheless supportive for a genetic component in its etiology. In the absence of chromosomal regions identified by linkage studies, research has focused on candidate gene approaches (by biological plausibility) rather than on positional approaches. Ancestral haplotype 8.1 as well as *TNF*, *IL10* and *SSA1* loci have been consistently associated with the disease although they are not specific for SS. In this review, the genetic component of SS is discussed on the basis of three known observations: (a) age at onset and sex-dependent presentation, (b) familial clustering of the disease, and (c) dissection of the genetic component. Since there is no strong evidence for a specific genetic component in SS, a large international and collaborative study would be suitable to assess the genetics of this disorder.

**Keywords:** *Sjögren's syndrome, major histocompatibility complex, IL10, SSA1, CHRM3*

### What is Sjögren's syndrome?

The immune system does not normally respond to self antigens, and consequently "normal" autoimmune responses are low and do not lead to pathological manifestations. This immunological tolerance was postulated over 50 years ago, but its multifactorial bases are still controversial (van Parijs et al. 1998). Tolerance is generated at two levels. The "upper level" of central tolerance develops primarily in fetal life, and the "lower level" of peripheral tolerance develops postnatally as a backup process. A faulty central tolerance sets the stage for AID, while faulty peripheral tolerance leads to overt manifestations. AID is a clinical syndrome thought to be caused by the loss of tolerance, and is characterized by activation of T cells or B cells, or both, leading to pathology in the absence of an ongoing infection or other discernible cause (Davidson and Diamond 2001). Nevertheless, it could be argued that a viral infection triggers the autoimmune response by direct cell injury, molecular mimicry, epitope spreading, bystander damage, and "viral déjà vu" mechanisms (Anaya et al. 2005b; Merkler et al. 2006). A key feature of immunology is that autoimmune response is physiologic and occurs in most persons, but pathologic autoimmunity (i.e. AID) develops in around 5% of the population (Cooper and Stroehla 2003). Thus, AID is the result of genetic, hormonal, immunological and environmental factors occurring simultaneously in an individual (Anaya et al. 2005b).

Sjögren's syndrome (SS) is a chronic AID characterized by a progressive lymphocytic and plasma cell infiltration of the salivary and lachrymal glands, accompanied by the production of autoantibodies leading to xerostomia and keratoconjunctivitis sicca (sicca-symptoms) (Anaya and Talal 1997). SS may occur alone (primary) or in association with other AID

Correspondence: J-M. Anaya, Corporación para Investigaciones Biológicas, Cra. 72-A No 78-B-141, Medellín, Colombia. Tel: 574 441 08 55. Fax: 57 441 55 14. E-mail: janaya@cib.org.co

ISSN 1740-2522 print/ISSN 1740-2530 online © 2006 Taylor & Francis
DOI: 10.1080/17402520600876911

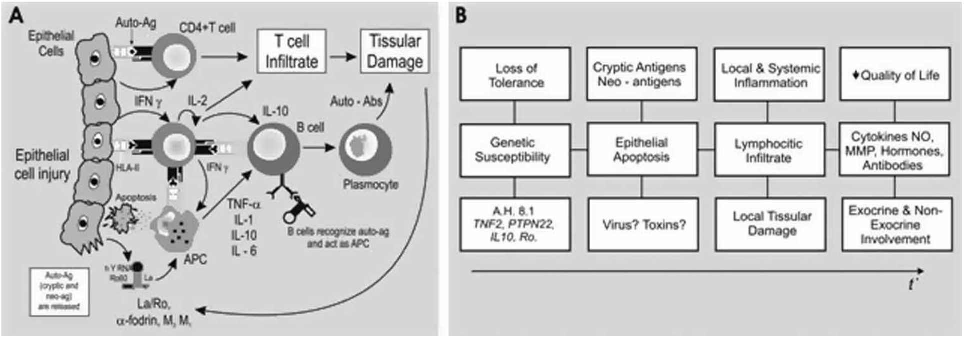

Figure 1. (A) Autoimmune response in primary SS. The epithelial cells (acinar and ductal) abnormally express HLA class II molecules and are thought to be antigen presenting cells (APC) to CD4+ T cells. IFN-γ induces HLA-class II, Fas and Bak expression, and participates in the apoptosis of epithelial cells, leading to the appearance of cryptic (auto) antigens (La, Ro, α-fodrin, muscarinic receptors). IL-10 is highly produced by CD4 T cells, and is responsible, together with IL-6, of B cell maturation and the synthesis of autoantibodies. IL-10 also participates in the T cells recruitment. Other inflammatory mediators (nitric oxide–NO–hormones, metalloproteinases-MMP) and cytokines are also released (i.e. IL-2, IL-1 and TNF-α), interfering with glandular function. (B) The three-stage-model of SS development. The disease is favored by gender and polymorphic genes (1st) that make epithelial cell susceptible to apoptosis (2nd, probably under an infectious or toxic insult), progressing then to salivary gland lymphocytic infiltration and subsequent production of autoantibodies and other soluble factors (3rd), all of which interfere with glandular function leading to the main clinical signs and symptoms.

(secondary), of which the most frequent are Hashimoto's thyroiditis and rheumatoid arthritis (RA). The spectrum of the disease may extend from an organ specific autoimmune disorder (autoimmune exocrinopathy) to a systemic process involving the musculoskeletal, pulmonary, gastrointestinal, hematological, vascular, dermatological, renal and nervous systems. Since the target tissue involved in the autoimmune histopathologic lesions of SS is the epithelium, the term "Autoimmune Epitheliitis" is currently used to describe the disorder (Moutsopoulos 1994). In the general population, the prevalence of primary SS ranges between 0.5 and 2.7% and is most common in the elderly (Anaya and Talal 1997; Thomas et al. 1998). This range may be attributed to the lack of uniform diagnostic criteria. Primary SS may occur in patients of all ages, but it mainly affects women (female:male ratio of 9:1) during the fourth and fifth decades of their lives (Anaya and Talal 1997). When SS occurs in children or men, its clinical and serological expressions are similar to the ones seen in adult women (Anaya et al. 1995a,b). Comparative studies between SS presentation in different ethnic populations indicate that the disease is distributed homogeneously in different parts of the world (Anaya and Talal 1997). Although SS may also be associated with an increased risk for B-cell lymphoma, the prevalence of such a complication is less than 5% of the cases and is reported more frequently in patients with primary SS than in those with secondary SS (Anaya et al. 1995a).

A key event in the initial process leading to primary SS seems to be increased epithelial cell apoptosis that progresses to subsequent salivary gland lymphocytic infiltration and autoantibody production (Humphreys-Beher et al. 1999; Mitsias et al. 2006). Immunohistologic analysis of lymphoid infiltrates in salivary gland tissue from patients with primary SS shows a high expression of human leukocyte antigen (HLA)-DR on acinar and ductal epithelial cells, suggesting that they may function as nonprofessional antigen-presenting cells and interact with CD4+ T cells (Fox et al. 1986). This interaction between epithelial and T cells leads to further production of cytokines and stimulation of B cell proliferation and differentiation. In addition, other soluble factors (i.e. nitric oxide) are released thus interfering with glandular function (Anaya et al. 2001) (Figure 1).

*Not all individuals with sicca-symptoms have SS*

The diagnosis of SS is based on the combination of symptoms (sicca-symptoms) and the presence of the autoimmune characteristics defined above: activation of T cells (i.e. positive salivary gland biopsy) or B cells (i.e. presence of "specific" autoantibodies). However, not all the individuals presenting sicca-symptoms have SS. Those individuals resembling SS but lacking the autoimmune criteria represent phenocopies (Anaya et al. 2001). No single test of oral or ocular involvement is sufficiently sensitive and specific to form a standard diagnosis of SS. Only the simultaneous positivity of various tests with the presence of subjective symptoms and serological abnormalities (anti-Ro and anti-La antibodies) and the presence of a score that is more than a "focus score" on the minor salivary gland (MSG) biopsy allow sufficient accuracy in the diagnosis of this disorder. Although no worldwide consensus exists as to the diagnostic criteria for SS, the Modified European classification criteria for SS have become very popular (Vitali et al. 2002). The demonstration of focal lymphocytic infiltrates on MSG biopsies has remained the gold standard for the oral component of SS. A cluster of at least 50 lymphocytes/4 $mm^2$ is called a "focus score"

(Daniels and Whitcher 1994). Multiple studies have shown that a positive MSG biopsy is closely correlated with keratoconjuntivitis sicca and anti-nuclear antibodies directed against Ro and La antigens (Daniels and Whitcher 1994).

### Genetic evidence for SS

Although the pathological mechanisms of AIDs remain poorly understood, a variety of studies have demonstrated that genetic predisposition is a major factor in disease susceptibility. In addition, given that these diseases hold a diverse group of phenotypes with overlapping features and that a tendency towards familial aggregation exists, it is likely that common underlying genes might be involved in AIDs (Criswell et al. 2005). In this review, the genetic component of SS is discussed on the basis of three observations: (a) age at onset and sex-dependent presentation, (b) familial clustering of the disease, and (c) dissection of the genetic component.

### Why is SS a female late-onset disease?

It has been postulated that environmental factors, such as hormonal influence, UV light, environmental exposures, and infectious agents play a role in the development of AIDs such as SS (James et al. 2001; Lockshin 2002), but these are still not completely understood, and need further research. Consideration of age as a factor contributing to the onset of AIDs at midlife (age, 40–60) has been proposed. However, detection bias could explain the apparently late-onset of disease because the progression of SS is slow to show signs and symptoms, making the age of onset imperceptible. In addition, age-dependent alteration of the biological functions may modify the development of the disease (i.e. decreased apoptosis and increased clonal activation of T cells, or decreased ability to respond to antigenic or mitogenic stimulation, etc.) (Hsu and Mountz 2003). Thus, the search for epigenetic factors influencing the triggering of SS is warranted.

The vast majority of patients with SS are female. The genetic reason behind this highly sex-related prevalence is poorly understood, although hormones like prolactin have been incriminated in the pathophysiology of the disease (Steinfeld et al. 2000; Taiym et al. 2004; Ackerman 2006). X-chromosome inactivation and the resultant tissue chimerism might be involved in the female predisposition for AIDs such as SS (Stewart 1999; Chitnis et al. 2000). Half the somatic cells in females express antigens derived from the paternal X and half from the maternal X. The Burnet–Jerne theory of somatic generation of antibody diversity and forbidden clone elimination states that lymphocytes under maturation in the thymus are killed or suppressed if they present high or low affinity towards a histocompatibility antigen. If this were to hold for self antigens as well, females would escape expressing one of their parental X chromosomes, which would still be able to react to self. Then, lymphocytes happening to pass the selection in the thymus would meet only cells expressing one of the parental X chromosomes. These would be more easily predisposed to a deregulation of self-tolerance in females than in males. This is known as the Kast Conjecture (Kast 1977). Even though specific responses to immunization do not appear to account for the high sex ratio seen in AIDs (Lockshin 2002), there is still a chance that chimerism among immunological cells could represent a starting point for perpetuating or acquiring an imbalance in self-tolerance. Ultimately, X monosomy as a resource for producing chromosome instability and also haploinsufficiency for X-linked genes have both been suggested as playing critical roles in the predominance of AIDs in females (Invernizzi et al. 2005). None of the proposals presented have a complete experimentally proven background and they are still part of a discussion on sex-connotation.

### Familial clustering of the disease

The powerful impact of genetic predisposition on susceptibility is usually based on disease concordance rates in monozygotic twins. Genetic contribution to AIDs is supported by the rates of monozygotic concordance, ranging from 15 to 60%, and by the high aggregation coefficients or recurrent risk ratios ($\lambda_R$) (Wandstrat and Wakeland 2001). However, genetic studies in primary SS twins have not been performed, and only a few case reports describing twins have been published (Simila et al. 1978; Besana et al. 1991; Ostuni et al. 1996; Houghton et al. 2005). The estimated disease concordance rate for identical twins would be on the lower-rank limit thus highlighting the importance of environmental factors in the susceptibility of SS. The prevalence of primary SS in siblings ($K_{SS}$) has been estimated to be 0.09% (Anaya et al. 2006b), while the reported general prevalence in Caucasians has been estimated to be $<0.1\%$ (Bowman et al. 2004). Therefore, genetic factors probably do not play a major role in the development of SS.

The observed aggregation of AIDs in families of patients with primary SS is nevertheless supportive for a genetic component in the etiology of the disease. Familial aggregation of autoimmune thyroid diseases (AITD), systemic lupus erythematosus (SLE), RA, diabetes mellitus type 1 (T1D), vitiligo, and all these AIDs taken together as a trait has been reported in families of primary SS patients (Block et al. 1975; Reveille et al. 1983; 1984; Molta et al. 1989; Foster et al. 1993a; Firooz et al. 1994; Ginn et al. 1998; Lin et al. 1998; Broadley et al. 2000; Michel et al. 2001; Prahalad et al. 2002; Sloka 2002; Alkhateeb et al. 2003;

Table I. HLA alleles associated with SS. Different HLA class II gene associations exhibited by populations of diverse ethnic origin. In addition, specific AAR or epitopes shared among these class II molecules and putatively involved in the binding and presentation of processed antigen to the TCR are shown, as well as alleles sharing amino acids but not associated with pSS. Single-letter amino acid code: G-glycine, A-alanine, V-valine, L-leucine, I-isoleucine, Y-tyrosine, W-tryptophan, H-histidine, K-lysine, R-arginine, Q-glutamine, E-glutamic acid, D-aspartic acid, S-serine, T-threonine.

| | HLA class II aleles associated with pSS | | | DQα | | | DQβ | | | | DRβ | | | | | | | |
|---|---|---|---|---|---|---|---|---|---|---|---|---|---|---|---|---|---|---|
| Population | HLA-DQA1 | HLA-DQB1 | HLA-DRB1 | 34 | 52 | 55 | 26 | 66 | 67 | 86 | 9 | 10 | 11 | 12 | 13 | 70 | 71 | 74 |
| Caucasoid* | DQA1*0501 | DQB1*0201 | DRB1*0301–0302 | Q | R | R | L | D | I | Q | E | Y | S | T | S | Q | K | R |
| Japanese† | DQA1*0301–0302 | DQB1*0401–0402 | DRB1*0405 | E | R | R | G | D | I | Q | E | Q | Y | K | H | Q | R | A |
| Chinese‡ | DQA1*0101–0103 | DQB1*0601 | DRB1*0803 | Q | S | G | Y | D | I | E | E | Y | S | T | G | D | R | L |
| Israeli Jewish and Greek Non-Jewish¶ | DQA1*0501 | DQB1*0301 | DRB1*1101,*1104 | Q | R | R | Y | E | V | Q | E | Y | S | T | M | D | R | A |
| Not associated with pSS | DQA1*0401 | DQB1*0602–0604 | DRB1*0404 | Q | R | R | L | E | V | E | E | Q | Y | K | H | Q | R | A |
| | DQA1*0601 | DQB1*0302–0303 | DRB1*1501–02 | Q | R | R | L | E | V | Q | W | Q | P | K | R | Q | A | A |

* Vitali et al. (1986), Kang et al. (1993), Kerttula et al. (1996), Bolstad et al. (2001), Anaya et al. (2002b, 2005a) and Gottenberg et al. (2003); † Kang et al. (1993) and Miyagawa et al. (1998); ‡ Kang et al. (1993); ¶ Papasteriades et al. (1988), Roitberg-Tambur et al. (1990) and Roitberg-Tambur et al. (1993).

Priori et al. 2003; Tait et al. 2004; Houghton et al. 2005; Anaya et al. 2006a,b). Other familial approximations have shown a greater frequency of positive Schimer's test, abnormal antibodies to thyroglobulin and elevated levels of gamma globulin on first-degree relatives of SS patients, with a transitional frequency among second-degree relatives (Bolstad et al. 2000a).

### Dissection of the genetic component

Segregation analyses have not been performed in SS. Current evidence indicates that as it occurs along with other AIDs, SS cannot be clearly classified according to a specific genetic Mendelian-like model (Bias et al. 1986; Anaya et al. 2005b). First, more than one gene seems to be involved in the development and outcome of the disease; therefore, the disease is polygenic. Second, the genes responsible that have been involved in its etiology do not represent rare variants, and are polymorphic. Third, pleiotropic interactions and possible epistasis may account for the development of SS (Wandstrat and Wakeland 2001).

The efforts to unravel the genetic component of SS have relied on association studies for disease gene identification (Tables II and III) However, robust analyses on candidate gene variants have not been undertaken and only a few linkage studies have been reported (Foster et al. 1993b). In the absence of chromosomal regions identified by linkage studies, research has focused on candidate gene approaches (by biological plausibility) rather than on positional approaches (Figure 1). As a result, several genes encoding molecules involved in apoptosis (*FAS*, *FASLG*), antigen processing and presentation (*HLA-DR*, *HLA-DQ*, *TAP1*, *TAP2*), immune recognition (*MBL2*, *IGHG*, *IGK*, *ABCA7/HA-1*), intercellular and intracellular signaling (*MMP9*, *CTLA4*, *PTPN22*), cytokine and chemokine transductional pathways (*IFNG*, *TGFB1*, *TNF*, *IL1B*, *IL1RN*, *IL13*, *IL4*, *IL6*, *IL10 CCR5*) and autoantigens (*SSA1*, *CHRM3*) have been reasonably tested in association studies (Tables I to III). However, little convincing replication and functional evidence exists. Additionally, given the patchy nature of linkage disequilibrium (LD) across the human genome (Gabriel et al. 2002), several associated polymorphisms would not have a causal role but could be associated with the disease due to another marker in its proximity. Furthermore, AID susceptibility is more likely to depend on a combination of quite subtle changes in the dynamic or expression of several genes, many of which could be present in the healthy population (Altshuler et al. 2000).

### Major histocompatibility complex and HLA associations

The major histocompatibility complex (MHC), the most important region in the human genome with

Table II. Non-HLA genetic association studies.

| Chromosomal band | Gene | Variant | Associated disease trait(s) | *P* value | OR | 95% IC | Case:control | Population | Reference |
|---|---|---|---|---|---|---|---|---|---|
| 1p13.3 | *PTPN22* | 1858T (620W) | Susceptibility | 0.01 | 2.42 | 1.24–4.75 | 70:308 | Colombian | Gomez et al. (2005) |
| 1q23 | *FASLG* | IVSnt-124A/G, IVS3nt169 T/- | | NS | | | 70:72 | Norwegian | Bolstad et al. (2000b) |
| 1q31–q32 | *IL10* | Hap-1082G-819C-592C | Susceptibility & early disease onset | NS | | | 108 cases | Australian | Limaye et al. (2000) |
| 1q31–q32 | *IL10* | Hap-1082-819-592 GCC/ATA | Susceptibility | $<0.05$ | 1.9 | 0.95–3.62 | 62 cases | Finnish | Hulkkonen et al. (2001a) |
| 1q31–q32 | *IL10* | Hap-1082G-819C-592C | Susceptibility & early disease onset | 0.006 & 0.034 | 5.3 & 5.5 | | 63:150 | Spanish | Font et al. (2002) |
| 1q31–q32 | *IL10* | IL-10.G9 | Cutaneous vasculitis & ↑ IL-10 serum levels | 0.04 & 0.02 | 5.3 & 5.5 | 1.2–2.4 & 1.1–2.2 | 39:15 | Colombian | Anaya et al. (2002a) |
| 1q31–q32 | *IL10* | Hap-1082G-819C-592C | Susceptibility | 0.003 | 2.25 | 1.26–4.02 | 129:96 | French | Gottenberg et al. (2004) |
| 2p12 | *IGK* | Ig KM | | NS | | | | Australian | Downie-Doyle et al. (2002) |
| 2p12 | *IGK* | Ig KM | Anti-La, disease activity | 0.016 | | | 65:66 | Finnish | Pertovaara et al. (2004) |
| 2q14 | *IL1B* | Hap-511C + 3953T | Susceptibility (protective) | 0.0006 | 0.43 | 0.25–0.74 | 69:392 | Colombian | Camargo et al. (2004) |
| 2q14 | *IL1B* | Hap-511CC-31TT 3877AA | Susceptibility | $<0.05$ | | | 101:105 | Japanese | Muraki et al. (2004) |
| 2q14.2 | *IL1RN* | ILRN*2 | Susceptibility & disease severity | $<0.05$ | | | | | Perrier et al. (1998) |
| 2q14.2 | *IL1RN* | VNTR | | NS | | | 39:76 | Slovak | Petrek et al. (2002) |
| 2q33 | *CTLA4* | (AT)n in 3′UTR | | NS | | | 58:150 | Tunisian | Hadj Kacem et al. (2001) |
| 2q33 | *CTLA4* | 49G | | NS | | | 58:150 | Tunisian | Hadj Kacem et al. (2001) |
| 3p21 | *CCR5* | CCR5wt/CCR5Δ32 heterozygosity | Susceptibility (protective) | 0.043 | 0.349 | 0.109–0.983 | 39:76 | Slovak | Petrek et al. (2002) |
| 5q31 | *IL13* | IL13 + 2044A/G | ↓ serum IgA and β2m ↓ Purpura | 0.030, 0.007 | | | 63:63 | Finnish | Pertovaara et al. (2006) |
| 5q31.1 | *IL4* | IL4-590 T/C | ↓ VSG | 0.010 | | | 63:63 | Finnish | Pertovaara et al. (2006) |
| 6p21.3 | *TAP1* | TAP1 + 333 + 637 | | NS | | | 74:76 | Colombian | (Anaya et al. 2005a) |
| 6p21.3 | *TAP1* | TAP1 + 333 + 637 | | NS | | | 57:80 | Colombian | Anaya et al. (2002b) |
| 6p21.3 | *TAP1* | Hap TNFa2 TAP*0101 TAP2*0101 | Susceptibility | 0.01 | 6.68 | | 45:130 | French | Jean et al. (1998) |
| 6p21.3 | *TAP2* | TAP2 + 379 + 565 + 665 | | NS | | | 74:76 | Colombian | Anaya et al. (2005a) |

Table II – *continued*

| Chromosomal band | Gene | Variant | Associated disease trait(s) | *P* value | OR | 95% IC | Case:control | Population | Reference |
|---|---|---|---|---|---|---|---|---|---|
| 6p21.3 | *TAP2* | TAP2 + 379 + 565 + 665 | | NS | | | 57:80 | Colombian | Anaya et al. (2002b) |
| 6p21.3 | *TAP2* | Hap TNFa2 TAP*0101 TAP2*0101 | Susceptibility | 0.01 | | | | French | Jean et al. (1998) |
| 6p21.3 | *TAP2* | *Bky2 (ATG → GTG; 577) Met → Val | Anti-Ro antibody production | 0.05 | | | 108 cases | Japanese | Kumagai et al. (1997) |
| 6p21.3 | *TNF* | TNF-308A | Susceptibility | 0.0001 | 2.9 | 1.90–4.57 | 67:430 | Colombian | Correa et al. (2005a) |
| 6p21.3 | *TNF* | TNF-308A | Susceptibility, anti-SSB/La | 0.00028 | 2.86 | 1.64–5.12 | 129:96 | French | Gottenberg et al. (2004) |
| 6p21.3 | *TNF* | TNFa2 | | NS | | | 58:150 | Tunisian | Hadj Kacem et al. (2001) |
| 6p21.3 | *TNF* | TNFa10 | | NS | | | | | Jean et al. (1998) |
| 6p21.3 | *TNF* | Hap TNFa2 TAP*0101 TAP2*0101 | Susceptibility | 0.01 | | | | | Jean et al. (1998) |
| 6p21.3 | *TNF* | TNF-308 | Clinical course & immunological features | NS | | | 65 patients | Colombian | Tobon et al. (2005) |
| 7p21 | *IL6* | −174C/G | ↑IL-6 plasma levels | $<0.05$ | | | 111:400 | Finnish | Hulkkonen et al. (2001b) |
| 7p21 | *IL6* | −174C/G | | NS | | | 129:96 | French | Gottenberg et al. (2004) |
| 10q11.2–q21 | *MBL2* | CGT → TGT;codon 52 | ↓histological grade and ↓MBL | 0.035 | 0.15 | 0.03–0.71 | 65:138 | Finland | Aittoniemi et al. (1996) |
| 10q11.2–q21 | *MBL2* | GGC → GAC;codon 54 | Susceptibility | $<0.05$ | | | | Japanese | Tsutsumi et al. (2001) |
| 10q24.1 | *FAS* | −671G, IVS2nt176C, IVS5nt82C | Susceptibility | 0.044, 0.030, 0.022 | 0.62, 1.71, 1.78 | 0.39–0.99, 1.05–2.80, 1.079–2.90 | 70:72 | Norwegian | Bolstad et al. (2000b) |
| 11p15.5 | *SSA1* | 7216A/G | Anti-SSA/Ro52 production | NS | | | 111:97 | Japanese | Imanishi et al. (2005) |
| 11p15.5 | *SSA1* | 7649A/G, 9571C/T | anti-Ro 52-kd–positive patients | 0.02, 0.00003 | | | 97:72 | Norwegian | Nakken et al. (2001) |
| 12q14 | *IFNG* | +874A/T | | NS | | | 129:96 | French | Gottenberg et al. (2004) |
| 14q32.33 | *IGHG* | GMz | Milder form of pSS | 0.004 | | | 65:66 | Finnish | Pertovaara et al. (2004) |
| 19p13.3 | *ABCA7* | | | NS | | | 94:545 | Norway, Hungary and Germany | Harangi et al. (2005) |
| 19p13.3 | *HA-1* | 500/504CA (168His) | Susceptibility | 0.003 | | | 94:545 | Norway, Hungary and Germany | Harangi et al. (2005) |

Table II – *continued*

| Chromosomal band | Gene | Variant | Associated disease trait(s) | *P* value | OR | 95% IC | Case:control | Population | Reference |
|---|---|---|---|---|---|---|---|---|---|
| 19q13.1 | *TGFB1* | C-509T | | NS | | | | North American | Caserta et al. (2004) |
| 19q13.2 | *TGFB1* | 869 C/T, 915 C/G | Anti-La | 0.0006 | 10.2 | 2.3–50.1 | 129:96 | French | Gottenberg et al. (2004) |
| 19q13.2 | *APOE* | epsilon4 allele | Early onset of pSS | 0.0407 | | | 63:64 | Finnish | Pertovaara et al. (2004) |
| 20q11.2–q13.1 | *MMP9* | 1562C → T | | NS | | | 66:66 | Finnish | Hulkkonen et al. (2004) |

*Genes abbreviations*: *ABCA7*, ATP-binding cassette, sub-family A (ABC1), member 7; *APOE*, apolipoprotein E (apoE); *CCR5*, chemokine (C–C motif) receptor 5; *CTLA4*, cytotoxic T-lymphocyte-associated protein 4; *FAS*, Fas (TNF receptor superfamily, member 6); *FASLG*, Fas ligand (TNF superfamily, member 6); *HA-1*, Minor hisocompatibility antigen; *IFNG*, interferon, gamma; *IGHG*, immunoglobulin heavy constant gamma; *IGK*, immunoglobulin kappa locus; *IL10*, interleukin 10; *IL13*, interleukin 13; *IL1B*, interleukin 1, beta; *IL1RN*, interleukin 1 receptor antagonist; *IL4*, interleukin 4; *IL6*, interleukin 6; *MBL2*, soluble mannose-binding lectin; *MMP9*, matrix metallopeptidase 9; *PTPN22*, protein tyrosine phosphatase, non-receptor type 22 (lymphoid); *SSA1*, Sjogren syndrome antigen A1 (52 kDa, ribonucleoprotein autoantigen SS-A/Ro); *TAP1*, transporter 1, ATP-binding cassette, sub-family B; *TAP2*, transporter 2, ATP-binding cassette, sub-family B; *TGFB1*, transforming growth factor, beta 1; *TNF*, tumor necrosis factor (TNF superfamily, member 2).

respect to adaptive and innate immune regulation, carries the major genetic influence on susceptibility to AIDs due to its highly polymorphic genes (Horton et al. 2004). The best identified genetic factors for primary SS are the MHC class II genes, mainly HLA-*DR* and HLA-*DQ* (Table I). HLA studies have two ultimate purposes: identify genetic prediction markers and provide new insights about the functional mechanisms underlying antigen presentation and the autoimmune response. As expected, patients with diverse ethnic origins carry different HLA susceptibility alleles (Mori et al. 2005), partly because immune-response gene polymorphisms have been shaped and naturally selected by population-specific histories of infectious diseases (Pearce and Merriman 2006). Comparisons between associated haplotypes have suggested critical *cis*- or *trans*-interaction of MHC protein segments that may provide the crucial conformation for peptide-binding and trigger specific CD4+ T cell responses (Kang et al. 1993). The β1 chain of HLA-DR molecules contains polymorphic residues which contribute five binding pockets: P1, P4, P6, P7 and P9. These pockets influence the peptide-binding specificity of different class II molecules. The P4 pocket, conformed by the amino acid residues (AAR) at positions β13, β70, β71 and β74, is critical for antigenic presentation (Stern et al. 1994). The last three AAR are in contact with the T cell receptor (TCR) and therefore, they are important in determining T cell recognition of the peptide–DR complex. The P4 pocket at the reported primary SS-susceptibility alleles across populations: DRB1*0301, *1101, *1104 and *0405 (Kang et al. 1993; Roitberg-Tambur et al. 1993), and DQB1 *0201 shares a common positively charged amino acid sequence (Gregersen et al. 1987; Anaya et al. 2005a). Having a positively charged P4 pocket implies that those positive HLA molecules can only present a neutral or negative peptide to T cells (Table I).

HLA-*DQB1* susceptibility alleles also share a region from AAR 59–69 located in the antigen-binding groove (Kang et al. 1993). An aspartic acid (D) and an isoleucine (I) at positions 66 and 67 are common AAR among DQB1*0201, *0401, *0402 and *0601 (Kang et al. 1993). Reveille et al. (1991) found that all primary SS patients with anti-Ro antibodies have a leucine (L) at position 26 of the HLA-DQB1 molecule and a glutamine (Q) at position 34 of the HLA-DQA1 molecule. Furthermore, a dose-dependent contribution of DQα-34Q and DQβ-26L, and the DRB1*03-DQB1*02-DQA1*0501 haplotype encompassing the shared DQβ-DI motif, might represent the strongest contributors to the formation of an anti-Ro/anti-La response in primary SS patients (Reveille et al. 1991).

Subsequent studies have found HLA class II alleles to be associated with specific subsets of autoantibodies rather than to the disease itself (Miyagawa et al. 1998;

Rischmueller et al. 1998; Gottenberg et al. 2003). Although selection bias could account for those results, HLA-DR2-DQA1*0102-DQB1*0602 (DR2-DQ1) haplotype has been found to be strongly associated with the presence of anti-Ro antibodies, whereas the risk of anti-Ro antibody spreading to produce precipitating anti-La antibodies might be higher in primary SS patients carrying the DR3-DQA1*0501-DQB1*02 (DR3-DQ2) haplotype (Rischmueller et al. 1998). Rischmueller et al. (1998) postulated that separate HLA class II associations reflect T cell recognition of unique epitopes derived from either or both of the La/Ro ribonucleoproteins, which might engender specific T helper responses thereby control diversification of the autoantibody response. Gottenberg et al. (2003) provided data supporting the epitope spreading hypothesis. They suggested that HLA-DR15 could favor anti-Ro synthesis while HLA-DR3 could favor both anti-Ro and anti-La production. On the other hand, the lack of association between HLA markers and clinical disease features indicates that HLA alleles do not predict clinical outcome (Gottenberg et al. 2003). However, both the heterogeneity of the disease and insufficient sample size are certainly obstacles to correct data analysis.

The haplotype HLA-DR3-DQ2 is part of the 8.1 ancestral haplotype (AH) (HLA-A1, C7, B8, C4AQ0, C4B1, DR3 and DQ2) which has been consistently associated with susceptibility to T1D, SLE, and other immunologic disorders (Price et al. 1999). Carriers of this 8.1 AH have an altered immune response characterized by an increased B cell function and by the synthesis of proinflammatory cytokines plus a decreased T cell response (Price et al. 1999). These immune abnormalities are also observed in patients with primary SS (Anaya et al. 1999).

### Non-HLA MHC genes

The extended MHC comprises an 8 Mb region at 6p21.3 and harbors approximately 200 genes, most of them coding for immunoregulatory molecules (Horton et al. 2004). *TAP1* and *TAP2* gene products are required for transporting and loading specific peptides to the MHC molecules (Tan et al. 1982). Kumagai et al. (1997) reported that TAP2*Bky2 (Val 577) allele was associated with the production of anti-Ro antibodies in Japanese. No association between *TAP* alleles and primary SS was found in the Colombian population (Anaya et al. 2002b). Studies considering both *TAP* and *HLA-DQB1* showed the existence of LD between them, suggesting a primary association caused by *HLA-DQB1* alleles or perhaps by the presence of another susceptibility gene located between them in this chromosome region (Anaya et al. 2002b). A mapping approach based on five microsatellites spanning 5 cM intervals within the MHC region predicted a new candidate region for acquiring primary SS, located at the most centromeric portion of the 6p21.31 chromosomal region (Anaya et al. 2003). One of the most likely genes related to this location appears to be *BAK1* (Herberg et al. 1998), which encodes a pro-apoptotic molecule belonging to the Bcl-2 protein family.

Tumor necrosis factor-alfa (TNFα) is encoded by the *TNF* gene, located within the class III region of the MHC, and is highly polymorphic. Five microsatellites and numerous single nucleotide polymorphisms (SNP) in the *TNF* promoter, some of which may regulate TNFα expression, have been described (Louis et al. 1998). Gottenberg et al. (2004) found an association between the −308A (TNF2) allele and SS patients positive for anti-La antibodies. We have observed that this allele is a common susceptibility factor in Colombians for primary SS, SLE and RA (Correa et al. 2005a,b). Studies of *TNF* microsatellite polymorphisms did not find any association in French (Guggenbuhl et al. 2000) nor in Tunisian populations (Hadj Kacem et al. 2001). Whether *TNF* association with SS is primary or secondary to LD with HLA-DRB1*03 and HLA-B8, is not clearly resolved yet (Wilson et al. 1993).

### Cytokine polymorphisms

Significantly higher serum cytokines and salivary levels of messenger RNA (mRNA) have been found in SS patients compared to control subjects for TNFα, interleukin (IL)-6, IL-10, interferon-gamma (IFNγ), and lower salivary gland expression of transforming growth factor beta1 (TGFβ1) (Fox et al. 1994; Koski et al. 1995; Ohyama et al. 1996). Although the extent to which these factors may contribute to the development and progression of primary SS remains to be elucidated (Magnusson et al. 2001), their polymorphic genes have been studied on the basis of functional SNPs, most of them at their promoter, may be related to protein expression (Table II).

IL-10 together with IL-6 plays a central role in the maturation of plasma cells and in the activation of immunoglobulin synthesis. In a murine model resembling SS, transgenic expression of IL-10 induced apoptosis of glandular tissue and lymphocyte infiltration consisting primarily of Fas-ligand (FasL)+ CD4+ T cells, as well as *in vitro* up-regulation of FasL expression on T cells (Saito et al. 1999). These findings resemble those observed in primary SS patients in whom increased production of IL-10 has been demonstrated by peripheral blood T cells (Villarreal et al. 1995), B cells, monocytes (Llorente et al. 1994), and also at the inflammatory site in MSG (Fox et al. 1994). The *IL10* gene is highly polymorphic. Two microsatellites and several SNPs have been reported (Hulkkonen et al. 2001a). Hulkkonen et al. (2001a) first reported an influence of haplotype GCC at positions −1082, −819 and

Table III. Genes encoding autoantigens investigated in SS.

| Chromosomal band | Gene | Variant | Associated trait(s) | *P* value | Case:control | Population | Reference |
|---|---|---|---|---|---|---|---|
| 1q43–44 | *CHRM3* | PCR-SSCP and AS | Anti-$M_3$ antibody production | NS | 70:140 | Colombian | Correa et al. (2005b) |
| 11p15.5 | *SSA1* | +7216 A/G | Anti-SSA/Ro52 production | NS | 111:97 | Japanese | Imanishi et al. (2005) |
| 11p15.5 | *SSA1* | +4595 C/T +7649 A/G +9571 C/T | Anti-Ro 52-kd–positive patients | 0.029 0.038 0.00003 | 97:72 | Norwegian | Nakken et al. (2001) |

*SSA1*, Sjogren syndrome antigen A1 (52 kDa, ribonucleoprotein autoantigen SS-A/Ro); *CHRM3*, cholinergic-receptor muscarinic 3; NS, statistically non-significative; PCR-SSCP, PCR-specific sequence conformational polymorphism; AS, automated sequencing.

−592 of *IL10* gene on susceptibility to primary SS in Finnish patients. Other studies have confirmed the influence of *IL10* locus on the disease (Limaye et al. 2000; Hulkkonen et al. 2001a; Font et al. 2002; Anaya et al. 2002a; Gottenberg et al. 2004) (Table II).

The IL-1 family consists of IL-1α, IL-1β, two receptors, and a specific IL-1 receptor antagonist (IL-1Ra) which inhibits the activity of IL-1α and IL-1β and modulates a variety of IL-1 related immune and inflammatory responses. Increased IL-1Ra serum levels in primary SS patients, as well as decreased salivary levels, suggest an important role for the local balance between IL-1 and IL-1Ra in the susceptibility to and severity of disease (Arend 2002). IL-1Ra is encoded by the *IL1RN* gene and a variable number of tandem repeats polymorphism within intron 2 has been shown to be a marker (*IL1RN*2* allele) for severe disease outcome (Perrier et al. 1998) (Table II).

### Autoantigens as candidate genes

One of the central clues to the pathogenesis of SS comes from the observation that the immune system targets a restricted and highly specific group of intracellular autoantigens which are ubiquitously expressed in many tissues. The Anti-Ro/SSA antibodies, commonly found in patients with SS and SLE, recognize the Ro/SSA ribonucleoprotein. The clustering and marked concentration of these molecules in the surface blebs of apoptotic cells, and their modification by apoptosis-specific proteolytic cleavage and other post-translational modifications, have focused attention on apoptosis as the potential initiating stimulus for systemic autoimmunity in SS (Rosen and Casciola-Rosen 2004). The Ro/SSA molecule is conformed by either a single 60- or 52-kD immunoreactive protein bound to 1 of 4 small RNA molecules (Itoh et al. 1991). Ro52 is coded by the *SSA1* gene located at 11p15.5 (Frank et al. 1993) and is thought to be a RING-finger-type E3 ubiquitin ligase (Wada and Kamitani 2006). The Ro60 gene (*SSA2*) maps to chromosome 1q31 (Frank and Mattei 1994) and it has been suggested that it works as part of a quality control of discard pathway for 5S ribosomal RNA (O'Brien and Wolin 1994). A third molecule with the properties of a Ro/SSA autoantigen is calreticulin, a 48-kD protein encoded by the *CALR* gene at 19p13.3–p13.2.

Nakken et al. (2001) found three SNPs in the *SSA1* gene associated with anti-Ro 52-kd autoantibodies in Caucasian primary SS patients (Table III). One of them +7649A/G SNP is located at a putative TATA box. Another +9571C/T SNP, associated with both anti-Ro 52-kD and anti-La antibodies, is located upstream of an alternatively spliced site, generating a shorter version of the protein (Ro52β). In contrast, Imanishi et al. (2005) identified one in the first intron, the +7216A/G SNP, this variant was not associated

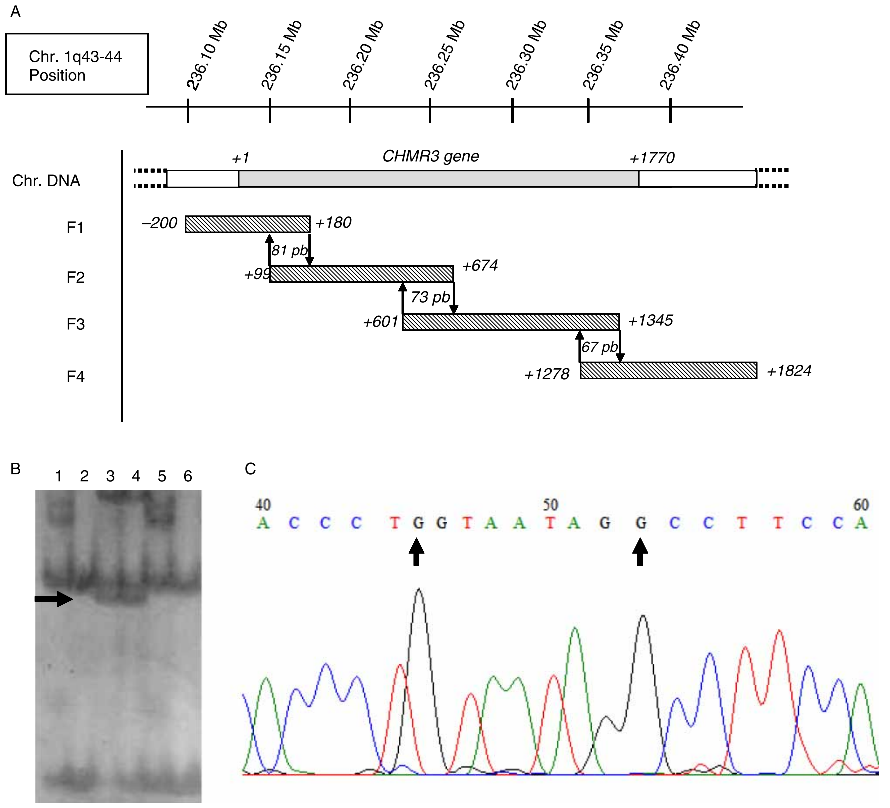

Figure 2. Coding region polymorphisms within the $M_3$ muscarinic-receptor gene (*CHMR3*). (A) Chromosomal position and size of the different obtained PCR fragments (F1–F4) used in the polymorphism screening of the *CHMR3*. The nucleotide numbering is shown for reference as well as overlapped nucleotides within fragments. (B) Sequence variation screening within 2024 bp of the *CHMR3* coding region. Wells 3 and 4 are mutation samples, the other wells are wild type sequences from studied individuals. (C) Sequencing results showing a single base pair deletion at positions +45 and +53. All sequences aligned perfectly with the published wild type Genbank sequence (NM_000740).

with the disease risk but with the presence of anti-SS-A/Ro52 antibody in patients with primary SS.

Transcript forms are known for La, another ribonucleoprotein target expressed in MSG tissue of patients with primary SS (Bachmann et al. 1996). A frame-shift mutation in exon 7 of the *SSB* gene results in a shorter version of La/SSB protein. The C-terminal region of La/SSB contains one of the major autoepitopes of this protein, and thus its modification might alter its antigenicity (Bachmann et al. 1996).

Auto-antibodies directed against muscarinic $M_3$ receptors may contribute to sicca symptoms and autonomic dysfunction in patients with both primary SS and secondary SS by inhibition of cholinergic neurotransmission at postsynaptic $M_3$ (Waterman et al. 2000; Goldblatt et al. 2002). The $M_3$ gene (*CHRM3*) is located at 1q43–44, and codes for a 590 amino acid protein. The gene is intronless (Fenech et al. 2001). Although the role of the anti-$M_3$ response is not clear, it has been suggested that these receptors might be synthesized in response to the generation of cryptic antigens that stimulate the activation of autoreactive T lymphocytes, consequently provoking an abnormal immune response and synthesis of antibodies (Rosen and Casciola-Rosen 2004). Considering that $M_3$ could be a possible target autoantigen in primary SS, our group performed a polymorphism screening in the gene coding region for *CHRM3* to test whether or not the gene would be incriminated in the risk of developing primary SS as has been observed in other AIDs in which polymorphisms in the autoantigen genes have been observed to be a risk factor for these diseases (Forsythe et al. 2002; Tomer and Davies 2003). Using the PCR-single specific conformational polymorphism technique and automated sequencing, only two simultaneous deletions, with a low frequency of 1.4% in the population under study were identified at the nucleotide positions +45 and +53 (Figure 2). Other studies have not been able to identify polymorphisms within the muscarinic coding or the flanking region (Fenech et al. 2001). As a corollary, the coding region of the *CHRM3* gene receptor is highly conserved and polymorphic variations within this region are unlikely to contribute to muscarinic receptor dysfunction in primary SS patients.

### Conclusion and perspectives

Although the clinical presentation and course of SS (i.e. phenotype) are similar among populations, the disease is fairly heterogeneous. This might be attributed to the effects of genotype (i.e. polymorphic genes) on phenotype under environmental or stochastic effects. From a genetic point of view, SS seems to be a complex disease, meaning that its inheritance does not follow a Mendelian-like model, and thus it is polygenic. However, the level of the genetic contribution to the disease is unknown. The AH 8.1 as well as *IL10* and *SSA1* loci have been consistently associated with the disease although they are not specific for SS. Aggregation of AIDs in families of patients with primary SS suggests that autoimmunity might be inherited as a trait rather than as a single phenotype (Anaya et al. 2006a,b).

The vast majority of association studies performed to date have been underpowered to detect the modest genetic effects that are responsible for common diseases (Plenge and Rioux 2006). Until recently, most studies have examined only a few SNPs in any candidate gene (which does not comprehensively test genetic variation in the gene), and most studies have examined only several tens of patients samples (which is underpowered to detect a true-positive association if the OR is modest (e.g. $<1.50$)) (Tables I–III). Human linkage studies of SS families, in addition to analyses on maternal transmission, imprinting, and X-chromosome inactivation will probably be an important starting place of information in the future.

Identification of genes that generate susceptibility to AIDs undoubtedly enhances our understanding of the mechanisms that mediate these complex diseases and will allow us to predict and prevent them as well as to discover new therapeutic interventions. Thus, a large international and collaborative study would be suitable to find conclusive evidence for a specific genetic component in SS.

### Acknowledgements

We thank the members of the Cellular Biology and Immunogenetic Unit for their fruitful discussions. Supported by Colciencias, Bogotá, Colombia (2213-04-11447).

### References

Ackerman LS. 2006. Sex hormones and the genesis of autoimmunity. Arch Dermatol 142:371–376.

Aittoniemi J, Miettinen A, Laippala P, Isolauri E, Viikari J, Ruuska T, Soppi E. 1996. Age-dependent variation in the serum concentration of mannan-binding protein. Acta Paediatr 85:906–909.

Alkhateeb A, Fain PR, Thody A, Bennett DC, Spritz RA. 2003. Epidemiology of vitiligo and associated autoimmune diseases in Caucasian probands and their families. Pigment Cell Res 16:208–214.

Altshuler D, Daly M, Kruglyak L. 2000. Guilt by association. Nat Genet 26:135–137.

Anaya JM, Liu GT, D'Souza E, Ogawa N, Luan X, Talal N. 1995a. Primary Sjogren's syndrome in men. Ann Rheum Dis 54:748–751.

Anaya JM, Ogawa N, Talal N. 1995b. Sjogren's syndrome in childhood. J Rheumatol 22:1152–1158.

Anaya JM, Talal N. 1997. Sjogren's syndrome and conective tissue diseases associated with immunologic disorders. In: Koopman W, editor. Athritis and allied conditions. 13th ed. Philadelphia: William & Wilkins. p 1561–1580.

Anaya JM, Correa PA, Mantilla RD. 1999. Síndrome de Sjögren primario. Características clínicas e inmunogenéticas. Acta Med Col :125–137.

Anaya JM, Ramos M, García M. 2001. Síndrome de Sjögren. Medellin: Corporación para Investigaciones Biológicas.

Anaya JM, Correa PA, Herrera M, Eskdale J, Gallagher G. 2002a. Interleukin 10 (IL-10) influences autoimmune response in primary Sjogren's syndrome and is linked to *IL-10* gene polymorphism. J Rheumatol 29:1874–1876.

Anaya JM, Correa PA, Mantilla RD, Arcos-Burgos M. 2002b. TAP, HLA-DQB1, and HLA-DRB1 polymorphism in Colombian patients with primary Sjogren's syndrome. Semin Arthritis Rheum 31:396–405.

Anaya JM, Rivera D, Palacio LG, Arcos-Burgos M, Correa PA. 2003. D6S439 microsatellite identifies a new susceptibility region for primary Sjogren's syndrome. J Rheumatol 30:2152–2156.

Anaya JM, Mantilla RD, Correa PA. 2005a. Immunogenetics of primary Sjogren's syndrome in Colombians. Semin Arthritis Rheum 34:735–743.

Anaya JM, Shoenfeld Y, Correa PA, Garcia-Carrasco M, Cervera R. 2005b. Autoimmunity and autoimmune disease. 1 ed. Medellin: CIB.

Anaya JM, Castiblanco J, Tobon GJ, Garcia J, Abad V, Cuervo H, Velasquez A, Angel ID, Vega P, Arango A. 2006a. Familial clustering of autoimmune diseases in patients with type 1 diabetes mellitus. J Autoimmun 26:208–214.

Anaya JM, Tobon GJ, Pineda-Tamayo R, Castiblanco J. 2006b. Autoimmune disease aggregation in families of patients with primary Sjögren's syndrome. J Rheumatol 33:2227–2234.

Arend WP. 2002. The balance between IL-1 and IL-1Ra in disease. Cytokine Growth Factor Rev 13:323–340.

Bachmann M, Hilker M, Grolz D, Tellmann G, Hake U, Kater L, de Wilde P, Troster H. 1996. Different La/SS-B mRNA isoforms are expressed in salivary gland tissue of patients with primary Sjogren's syndrome. J Autoimmun 9:757–766.

Besana C, Salmaggi C, Pellegrino C, Pierro L, Vergani S, Faravelli A, Rugarli C. 1991. Chronic bilateral dacryo-adenitis in identical twins: A possible incomplete form of Sjogren syndrome. Eur J Pediatr 150:652–655.

Bias WB, Reveille JD, Beaty TH, Meyers DA, Arnett FC. 1986. Evidence that autoimmunity in man is a mendelian dominant trait. Am J Hum Genet :584–602.

Block SR, Winfield JB, Lockshin MD, D'Angelo WA, Christian CL. 1975. Studies of twins with systemic lupus erythematosus. A review of the literature and presentation of 12 additional sets. Am J Med 59:533–552.

Bolstad AI, Haga HJ, Wassmuth R, Jonsson R. 2000a. Monozygotic twins with primary Sjogren's syndrome. J Rheumatol 27:2264–2266.

Bolstad AI, Wargelius A, Nakken B, Haga HJ, Jonsson R. 2000b. Fas and Fas ligand gene polymorphisms in primary Sjogren's syndrome. J Rheumatol 27:2397–2405.

Bolstad AI, Wassmuth R, Haga HJ, Jonsson R. 2001. HLA markers and clinical characteristics in Caucasians with primary Sjogren's syndrome. J Rheumatol 28:1554–1562.

Bowman SJ, Ibrahim GH, Holmes G, Hamburger J, Ainsworth JR. 2004. Estimating the prevalence among Caucasian women of primary Sjogren's syndrome in two general practices in Birmingham, UK. Scand J Rheumatol 33:39–43.

Broadley SA, Deans J, Sawcer SJ, Clayton D, Compston DA. 2000. Autoimmune disease in first-degree relatives of patients with multiple sclerosis. A UK survey. Brain 123(Pt 6):1102–1111.

Camargo JF, Correa PA, Castiblanco J, Anaya JM. 2004. Interleukin-1beta polymorphisms in Colombian patients with autoimmune rheumatic diseases. Genes Immun 5:609–614.

Caserta TM, Knisley AA, Tan FK, Arnett FC, Brown TL. 2004. Genotypic analysis of the TGF beta-509 allele in patients with systemic lupus erythematosus and Sjogren's syndrome. Ann Genet 47:359–363.

Cooper GS, Stroehla BC. 2003. The epidemiology of autoimmune diseases. Autoimmun Rev 2:119–125.

Correa PA, Gomez LM, Cadena J, Anaya JM. 2005a. Autoimmunity and tuberculosis. Opposite association with TNF polymorphism. J Rheumatol 32:219–224.

Correa PA, Sterin-Borda L, Borda E, Anaya JM. 2005b. Cholinergic M3 receptor gene polymorhism in primary Sjogren's syndrome. Ann Rheum Dis 64(Suppl 3):116.

Chitnis S, Monteiro J, Glass D, Apatoff B, Salmon J, Concannon P, Gregersen PK. 2000. The role of X-chromosome inactivation in female predisposition to autoimmunity. Arthritis Res 2:399–406.

Criswell LA, Pfeiffer KA, Lum RF, Gonzales B, Novitzke J, Kern M, Moser KL, Begovich AB, Carlton VE, Li W, et al. 2005. Analysis of families in the multiple autoimmune disease genetics consortium (MADGC) collection: The PTPN22 620 W allele associates with multiple autoimmune phenotypes. Am J Hum Genet 76:561–571.

Daniels TE, Whitcher JP. 1994. Association of patterns of labial salivary gland inflammation with keratoconjunctivitis sicca. Analysis of 618 patients with suspected Sjogren's syndrome. Arthritis Rheum 37:869–877.

Davidson A, Diamond B. 2001. Autoimmune diseases. N Engl J Med 345:340–350.

Downie-Doyle S, Lester S, Bardy P, Gordon T, Rischmueller M, Pile K. 2002. Immunoglobulin kappa light chain gene alleles are not associated with primary Sjogren's syndrome. Genes Immun 1(3 Suppl):S63–S65.

Fenech AG, Ebejer MJ, Felice AE, Ellul-Micallef R, Hall IP. 2001. Mutation screening of the muscarinic M(2) and M(3) receptor genes in normal and asthmatic subjects. Br J Pharmacol 133:43–48.

Firooz A, Mazhar A, Ahmed AR. 1994. Prevalence of autoimmune diseases in the family members of patients with pemphigus vulgaris. J Am Acad Dermatol 31:434–437.

Font J, Garcia-Carrasco M, Ramos-Casals M, Aldea AI, Cervera R, Ingelmo M, Vives J, Yague J. 2002. The role of interleukin-10 promoter polymorphisms in the clinical expression of primary Sjogren's syndrome. Rheumatology (Oxford) 41:1025–1030.

Forsythe SM, Kogut PC, McConville JF, Fu Y, McCauley JA, Halayko AJ, Liu HW, Kao A, Fernandes DJ, Bellam S, et al. 2002. Structure and transcription of the human m3 muscarinic receptor gene. Am J Respir Cell Mol Biol 26:298–305.

Foster H, Fay A, Kelly C, Charles P, Walker D, Griffiths I. 1993a. Thyroid disease and other autoimmune phenomena in a family study of primary Sjogren's syndrome. Br J Rheumatol 32:36–40.

Foster H, Stephenson A, Walker D, Cavanagh G, Kelly C, Griffiths I. 1993b. Linkage studies of HLA and primary Sjogren's syndrome in multicase families. Arthritis Rheum 36:473–484.

Fox RI, Bumol T, Fantozzi R, Bone R, Schreiber R. 1986. Expression of histocompatibility antigen HLA-DR by salivary gland epithelial cells in Sjogren's syndrome. Arthritis Rheum 29:1105–1111.

Fox RI, Kang HI, Ando D, Abrams J, Pisa E. 1994. Cytokine mRNA expression in salivary gland biopsies of Sjogren's syndrome. J Immunol 152:5532–5539.

Frank MB, Itoh K, Fujisaku A, Pontarotti P, Mattei MG, Neas BR. 1993. The mapping of the human 52-kD Ro/SSA autoantigen gene to human chromosome 11, and its polymorphisms. Am J Hum Genet 52:183–191.

Frank MB, Mattei MG. 1994. Mapping of the human 60,000 M(r) Ro/SSA locus: The genes for three Ro/SSA autoantigens are located on separate chromosomes. Immunogenetics 39: 428–431.

Gabriel SB, Schaffner SF, Nguyen H, Moore JM, Roy J, Blumenstiel B, Higgins J, DeFelice M, Lochner A, Faggart M, et al. 2002. The structure of haplotype blocks in the human genome. Science 296:2225–2229.

Ginn LR, Lin JP, Plotz PH, Bale SJ, Wilder RL, Mbauya A, Miller FW. 1998. Familial autoimmunity in pedigrees of idiopathic inflammatory myopathy patients suggests common genetic risk factors for many autoimmune diseases. Arthritis Rheum 41:400–405.

Goldblatt F, Gordon TP, Waterman SA. 2002. Antibody-mediated gastrointestinal dysmotility in scleroderma. Gastroenterology 123:1144–1150.

Gomez LM, Anaya JM, Gonzalez CI, Pineda-Tamayo R, Otero W, Arango A, Martin J. 2005. PTPN22 C1858T polymorphism in Colombian patients with autoimmune diseases. Genes Immun 6:628–631.

Gottenberg JE, Busson M, Loiseau P, Cohen-Solal J, Lepage V, Charron D, Sibilia J, Mariette X. 2003. In primary Sjogren's syndrome, HLA class II is associated exclusively with autoantibody production and spreading of the autoimmune response. Arthritis Rheum 48:2240–2245.

Gottenberg JE, Busson M, Loiseau P, Dourche M, Cohen-Solal J, Lepage V, Charron D, Miceli C, Sibilia J, Mariette X. 2004. Association of transforming growth factor beta1 and tumor necrosis factor alpha polymorphisms with anti-SSB/La antibody secretion in patients with primary Sjogren's syndrome. Arthritis Rheum 50:570–580.

Gregersen PK, Silver J, Winchester RJ. 1987. The shared epitope hypothesis. An approach to understanding the molecular genetics of susceptibility to rheumatoid arthritis. Arthritis Rheum 30:1205–1213.

Guggenbuhl P, Veillard E, Quelvenec E, Jego P, Semana G, Jean S, Meadeb J, Chales G, Perdriger A. 2000. Analysis of TNFalpha microsatellites in 35 patients with primary Sjogren's syndrome. Joint Bone Spine 67:290–295.

Hadj Kacem H, Kaddour N, Adyel FZ, Bahloul Z, Ayadi H. 2001. HLA-DQB1 CAR1/CAR2, TNFa IR2/IR4 and CTLA-4 polymorphisms in Tunisian patients with rheumatoid arthritis and Sjogren's syndrome. Rheumatology (Oxford) 40: 1370–1374.

Harangi M, Kaminski WE, Fleck M, Orso E, Zeher M, Kiss E, Szekanecz Z, Zilahi E, Marienhagen J, Aslanidis C, et al. 2005. Homozygosity for the 168His variant of the minor histocompatibility antigen HA-1 is associated with reduced risk of primary Sjogren's syndrome. Eur J Immunol 35:305–317.

Herberg JA, Phillips S, Beck S, Jones T, Sheer D, Wu JJ, Prochazka V, Barr PJ, Kiefer MC, Trowsdale J. 1998. Genomic structure and domain organisation of the human Bak gene. Gene 211:87–94.

Horton R, Wilming L, Rand V, Lovering RC, Bruford EA, Khodiyar VK, Lush MJ, Povey S, Talbot CC Jr, Wright MW, et al. 2004. Gene map of the extended human MHC. Nat Rev Genet 5:889–899.

Houghton KM, Cabral DA, Petty RE, Tucker LB. 2005. Primary Sjogren's syndrome in dizygotic adolescent twins: One case with lymphocytic interstitial pneumonia. J Rheumatol 32: 1603–1606.

Hsu HC, Mountz JD. 2003. Origin of late-onset autoimmune disease. Immunol Allergy Clin North Am 23(vi):65–82.

Hulkkonen J, Pertovaara M, Antonen J, Lahdenpohja N, Pasternack A, Hurme M. 2001a. Genetic association between interleukin-10 promoter region polymorphisms and primary Sjogren's syndrome. Arthritis Rheum 44:176–179.

Hulkkonen J, Pertovaara M, Antonen J, Pasternack A, Hurme M. 2001b. Elevated interleukin-6 plasma levels are regulated by the promoter region polymorphism of the *IL6* gene in primary

Sjogren's syndrome and correlate with the clinical manifestations of the disease. Rheumatology (Oxford) 40:656–661.

Hulkkonen J, Pertovaara M, Antonen J, Pasternack A, Hurme M, Pollanen P, Lehtimaki T. 2004. Matrix metalloproteinase 9 (*MMP-9*) gene polymorphism and MMP-9 plasma levels in primary Sjogren's syndrome. Rheumatology (Oxford) 43: 1476–1479.

Humphreys-Beher MG, Peck AB, Dang H, Talal N. 1999. The role of apoptosis in the initiation of the autoimmune response in Sjogren's syndrome. Clin Exp Immunol 116:383–387.

Imanishi T, Morinobu A, Hayashi N, Kanagawa S, Koshiba M, Kondo S, Kumagai S. 2005. A novel polymorphism of the *SSA1* gene is associated with anti-SS-A/Ro52 autoantibody in Japanese patients with primary Sjogren's syndrome. Clin Exp Rheumatol 23:521–524.

Invernizzi P, Miozzo M, Selmi C, Persani L, Battezzati PM, Zuin M, Lucchi S, Meroni PL, Marasini B, Zeni S, et al. 2005. X chromosome monosomy: A common mechanism for autoimmune diseases. J Immunol 175:575–578.

Itoh K, Itoh Y, Frank MB. 1991. Protein heterogeneity in the human Ro/SSA ribonucleoproteins. The 52- and 60-kD Ro/SSA autoantigens are encoded by separate genes. J Clin Invest 87:177–186.

James JA, Harley JB, Scofield RH. 2001. Role of viruses in systemic lupus erythematosus and Sjogren syndrome. Curr Opin Rheumatol 13:370–376.

Jean S, Quelvennec E, Alizadeh M, Guggenbuhl P, Birebent B, Perdriger A, Grosbois B, Pawlotsky PY, Semana G. 1998. DRB1*15 and DRB1*03 extended haplotype interaction in primary Sjogren's syndrome genetic susceptibility. Clin Exp Rheumatol 16:725–728.

Kang HI, Fei HM, Saito I, Sawada S, Chen SL, Yi D, Chan E, Peebles C, Bugawan TL, Erlich HA, et al. 1993. Comparison of HLA class II genes in Caucasoid Chinese, and Japanese patients with primary Sjogren's syndrome. J Immunol 150:3615–3623.

Kast RE. 1977. Predominance of autoimmune and rheumatic diseases in females. J Rheumatol 4:288–292.

Kerttula TO, Collin P, Polvi A, Korpela M, Partanen J, Maki M. 1996. Distinct immunologic features of Finnish Sjogren's syndrome patients with HLA alleles DRB1*0301, DQA1*0501, and DQB1*0201. Alterations in circulating T cell receptor gamma/delta subsets. Arthritis Rheum 39: 1733–1739.

Koski H, Konttinen YT, Gu XH, Hietanen J, Malmstrom M. 1995. Transforming growth factor beta 2 in labial salivary glands in Sjogren's syndrome. Ann Rheum Dis 54:744–747.

Kumagai S, Kanagawa S, Morinobu A, Takada M, Nakamura K, Sugai S, Maruya E, Saji H. 1997. Association of a new allele of the *TAP2* gene, TAP2*Bky2 (Val577), with susceptibility to Sjogren's syndrome. Arthritis Rheum 40:1685–1692.

Limaye V, Lester S, Downie-Doyle S, Pile K, Bardy P, Gordon TP, Rischmueller M. 2000. Polymorphisms of the interleukin 10 gene promoter are not associated with anti-Ro autoantibodies in primary Sjogren's syndrome. J Rheumatol 27:2945–2946.

Lin JP, Cash JM, Doyle SZ, Peden S, Kanik K, Amos CI, Bale SJ, Wilder RL. 1998. Familial clustering of rheumatoid arthritis with other autoimmune diseases. Hum Genet 103:475–482.

Lockshin MD. 2002. Sex ratio and rheumatic disease. Autoimmun Rev 1:162–167.

Louis E, Franchimont D, Piron A, Gevaert Y, Schaaf-Lafontaine N, Roland S, Mahieu P, Malaise M, De Groote D, Louis R, Belaiche J. 1998. Tumour necrosis factor (TNF) gene polymorphism influences TNF-alpha production in lipopoly-saccharide (LPS)-stimulated whole blood cell culture in healthy humans. Clin Exp Immunol 113:401–406.

Llorente L, Richaud-Patin Y, Fior R, Alcocer-Varela J, Wijdenes J, Fourrier BM, Galanaud P, Emilie D. 1994. *In vivo* production of interleukin-10 by non-T cells in rheumatoid arthritis. Sjogren's syndrome, and systemic lupus erythematosus. A potential mechanism of B lymphocyte hyperactivity and autoimmunity. Arthritis Rheum 37:1647–1655.

Magnusson V, Nakken B, Bolstad AI, Alarcon-Riquelme ME. 2001. Cytokine polymorphisms in systemic lupus erythematosus and Sjogren's syndrome. Scand J Immunol 54:55–61.

Merkler D, Horvath E, Bruck W, Zinkernagel RM, Del la Torre JC, Pinschewer DD. 2006. "Viral deja vu" elicits organ-specific immune disease independent of reactivity to self. J Clin Invest 116:1254–1263.

Michel M, Johanet C, Meyer O, Frances C, Wittke F, Michel C, Arfi S, Tournier-Lasserve E, Piette JC. 2001. Familial lupus erythematosus. Clinical and immunologic features of 125 multiplex families. Medicine (Baltimore) 80:153–158.

Mitsias DI, Kapsogeorgou EK, Moutsopoulos HM. 2006. The role of epithelial cells in the initiation and perpetuation of autoimmune lesions: Lessons from Sjögren's syndrome (auto-immune epithelitis). Lupus 15:255–261.

Miyagawa S, Shinohara K, Nakajima M, Kidoguchi K, Fujita T, Fukumoto T, Yoshioka A, Dohi K, Shirai T. 1998. Polymorph-isms of HLA class II genes and autoimmune responses to Ro/SS-A-La/SS-B among Japanese subjects. Arthritis Rheum 41:927–934.

Molta CT, Khan MA, Aponte CJ, Reynolds TL, Macintyre SS. 1989. Familial occurrence of systemic sclerosis, rheumatoid arthritis and other immunological disorders: Report of two kindreds with study of HLA antigens and review of the literature. Clin Exp Rheumatol 7:229–236.

Mori M, Yamada R, Kobayashi K, Kawaida R, Yamamoto K. 2005. Ethnic differences in allele frequency of autoimmune-disease-associated SNPs. J Hum Genet 50:264–266.

Moutsopoulos HM. 1994. Sjogren's syndrome: Autoimmune epithelitis. Clin Immunol Immunopathol 72:162–165.

Muraki Y, Tsutsumi A, Takahashi R, Suzuki E, Hayashi T, Chino Y, Goto D, Matsumoto I, Murata H, Noguchi E, Sumida T. 2004. Polymorphisms of *IL-1 beta* gene in Japanese patients with Sjogren's syndrome and systemic lupus erythematosus. J Rheumatol 31:720–725.

Nakken B, Jonsson R, Bolstad AI. 2001. Polymorphisms of the *Ro52* gene associated with anti-Ro 52-kd autoantibodies in patients with primary Sjogren's syndrome. Arthritis Rheum 44:638–646.

O'Brien CA, Wolin SL. 1994. A possible role for the 60-kD Ro autoantigen in a discard pathway for defective 5S rRNA precursors. Genes Dev 8:2891–2903.

Ohyama Y, Nakamura S, Matsuzaki G, Shinohara M, Hiroki A, Fujimura T, Yamada A, Itoh K, Nomoto K. 1996. Cytokine messenger RNA expression in the labial salivary glands of patients with Sjogren's syndrome. Arthritis Rheum 39: 1376–1384.

Ostuni PA, Ianniello A, Sfriso P, Mazzola G, Andretta M, Gambari PF. 1996. Juvenile onset of primary Sjogren's syndrome: Report of 10 cases. Clin Exp Rheumatol 14:689–693.

Papasteriades CA, Skopouli FN, Drosos AA, Andonopoulos AP, Moutsopoulos HM. 1988. HLA-alloantigen associations in Greek patients with Sjogren's syndrome. J Autoimmun 1:85–90.

Pearce SH, Merriman TR. 2006. Genetic progress towards the molecular basis of autoimmunity. Trends Mol Med 12:90–98.

Perrier S, Coussediere C, Dubost JJ, Albuisson E, Sauvezie B. 1998. IL-1 receptor antagonist (*IL-1RA*) gene polymorphism in Sjogren's syndrome and rheumatoid arthritis. Clin Immunol Immunopathol 87:309–313.

Pertovaara M, Lehtimaki T, Rontu R, Antonen J, Pasternack A, Hurme M. 2004. Presence of apolipoprotein E epsilon4 allele predisposes to early onset of primary Sjogren's syndrome. Rheumatology (Oxford) 43:1484–1487.

Pertovaara M, Antonen J, Hurme M. 2006. Th2 cytokine genotypes are associated with a milder form of primary Sjogren's syndrome. Ann Rheum Dis 65:666–670.

Petrek M, Cermakova Z, Hutyrova B, Micekova D, Drabek J, Rovensky J, Bosak V. 2002. CC chemokine receptor 5 and interleukin-1 receptor antagonist gene polymorphisms in patients with primary Sjogren's syndrome. Clin Exp Rheumatol 20:701–703.

Plenge R, Rioux JD. 2006. Identifying susceptibility genes for immunological disorders: Patterns, power, and proof. Immunol Rev 210:40–51.

Prahalad S, Shear ES, Thompson SD, Giannini EH, Glass DN. 2002. Increased prevalence of familial autoimmunity in simplex and multiplex families with juvenile rheumatoid arthritis. Arthritis Rheum 46:1851–1856.

Price P, Witt C, Allcock R, Sayer D, Garlepp M, Kok CC, French M, Mallal S, Christiansen F. 1999. The genetic basis for the association of the 8.1 ancestral haplotype (A1, B8, DR3) with multiple immunopathological diseases. Immunol Rev 167:257–274.

Priori R, Medda E, Conti F, Cassara EA, Danieli MG, Gerli R, Giacomelli R, Franceschini F, Manfredi A, Pietrogrande M, et al. 2003. Familial autoimmunity as a risk factor for systemic lupus erythematosus and vice versa: A case-control study. Lupus 12:735–740.

Reveille JD, Bias WB, Winkelstein JA, Provost T, Dorsch CA, Arnett FC. 1983. Familial systemic lupus erythematosus: Immunogenetic studies in eight families. Medicine (Baltimore) 1:21–35.

Reveille JD, Wilson RW, Provost TT, Bias WB, Arnett FC. 1984. Primary Sjogren's syndrome and other autoimmune diseases in families. Prevalence and immunogenetic studies in six kindreds. Ann Intern Med 101:748–756.

Reveille JD, Macleod MJ, Whittington K, Arnett FC. 1991. Specific amino acid residues in the second hypervariable region of HLA-DQA1 and DQB1 chain genes promote the Ro (SS-A)/La (SS-B) autoantibody responses. J Immunol 146:3871–3876.

Rischmueller M, Lester S, Chen Z, Champion G, Van Den Berg R, Beer R, Coates T, McCluskey J, Gordon T. 1998. HLA class II phenotype controls diversification of the autoantibody response in primary Sjogren's syndrome (pSS). Clin Exp Immunol 111:365–371.

Roitberg-Tambur A, Brautbar C, Markitziu A, Ben-Chetrit E, Rubinow A, Friedmann A. 1990. Immunogenetics of HLA class II genes in primary Sjogren's syndrome in Israeli Jewish patients. Isr J Med Sci 26:677–681.

Roitberg-Tambur A, Friedmann A, Safirman C, Markitziu A, Ben-Chetrit E, Rubinow A, Moutsopoulos HM, Stavropoulos E, Skopouli FN, Margalit H, et al. 1993. Molecular analysis of HLA class II genes in primary Sjogren's syndrome. A study of Israeli Jewish and Greek non-Jewish patients. Hum Immunol 36:235–242.

Rosen A, Casciola-Rosen L. 2004. Altered autoantigen structure in Sjogren's syndrome: Implications for the pathogenesis of autoimmune tissue damage. Crit Rev Oral Biol Med 15: 156–164.

Saito I, Haruta K, Shimuta M, Inoue H, Sakurai H, Yamada K, Ishimaru N, Higashiyama H, Sumida T, Ishida H, et al. 1999. Fas ligand-mediated exocrinopathy resembling Sjogren's syndrome in mice transgenic for IL-10. J Immunol 162:2488–2494.

Simila S, Kokkonen J, Kaski M. 1978. Achalasia sicca—juvenile Sjogren's syndrome with achalasia and gastric hyposecretion. Eur J Pediatr 129:175–181.

Sloka S. 2002. Observations on recent studies showing increased co-occurrence of autoimmune diseases. J Autoimmun 18:251–257.

Steinfeld S, Maho A, Chaboteaux C, Daelemans P, Pochet R, Appelboom T, Kiss R. 2000. Prolactin up-regulates cathepsin B and D expression in minor salivary glands of patients with Sjogren's syndrome. Lab Invest 80:1711–1720.

Stern LJ, Brown JH, Jardetzky TS, Gorga JC, Urban RG, Strominger JL, Wiley DC. 1994. Crystal structure of the human class II MHC protein HLA-DR1 complexed with an influenza virus peptide. Nature 368:215–221.

Stewart JJ. 1999. Theory and treatment of the X-inactivation chimera in female-prevalent autoimmune disease. Arch Immunol Ther Exp (Warsz) 47:355–359.

Tait KF, Marshall T, Berman J, Carr-Smith J, Rowe B, Todd JA, Bain SC, Barnett AH, Gough SC. 2004. Clustering of autoimmune disease in parents of siblings from the type 1 diabetes Warren repository. Diabet Med 21:358–362.

Taiym S, Haghighat N, Al-Hashimi I. 2004. A comparison of the hormone levels in patients with Sjogren's syndrome and healthy controls. Oral Surg Oral Med Oral Pathol Oral Radiol Endod 97:579–583.

Tan EM, Cohen AS, Fries JF, Masi AT, McShane DJ, Rothfield NF, Schaller JG, Talal N, Winchester RJ. 1982. The 1982 revised criteria for the classification of systemic lupus erythematosus. Arthritis Rheum 25:1271–1277.

Thomas E, Hay EM, Hajeer A, Silman AJ. 1998. Sjogren's syndrome: A community-based study of prevalence and impact. Br J Rheumatol 37:1069–1076.

Tobon GJ, Correa PA, Gomez LM, Anaya JM. 2005. Lack of association between TNF-308 polymorphism and the clinical and immunological characteristics of systemic lupus erythematosus and primary Sjogren's syndrome. Clin Exp Rheumatol 23:339–344.

Tomer Y, Davies TF. 2003. Searching for the autoimmune thyroid disease susceptibility genes: From gene mapping to gene function. Endocr Rev 24:694–717.

Tsutsumi A, Sasaki K, Wakamiya N, Ichikawa K, Atsumi T, Ohtani K, Suzuki Y, Koike T, Sumida T. 2001. Mannose-binding lectin gene: Polymorphisms in Japanese patients with systemic lupus erythematosus, rheumatoid arthritis and Sjogren's syndrome. Genes Immun 2:99–104.

van Parijs L, Perez VL, Abbas AK. 1998. Mechanisms of peripheral T cell tolerance. Novartis Found Symp 215:5–14; discussion 14–20, 33–40.

Villarreal GM, Alcocer-Varela J, Llorente L. 1995. Cytokine gene and CD25 antigen expression by peripheral blood T cells from patients with primary Sjogren's syndrome. Autoimmunity 20:223–229.

Vitali C, Tavoni A, Rizzo G, Neri R, D'Ascanio A, Cristofani R, Bombardieri S. 1986. HLA antigens in Italian patients with primary Sjogren's syndrome. Ann Rheum Dis 45:412–416.

Vitali C, Bombardieri S, Jonsson R, Moutsopoulos HM, Alexander EL, Carsons SE, Daniels TE, Fox PC, Fox RI, Kassan SS, et al. 2002. Classification criteria for Sjogren's syndrome: A revised version of the European criteria proposed by the American–European Consensus Group. Ann Rheum Dis 61:554–558.

Wada K, Kamitani T. 2006. Autoantigen Ro52 is an E3 ubiquitin ligase. Biochem Biophys Res Commun 339:415–421.

Wandstrat A, Wakeland E. 2001. The genetics of complex autoimmune diseases: Non-MHC susceptibility genes. Nat Immunol 2:802–809.

Waterman SA, Gordon TP, Rischmueller M. 2000. Inhibitory effects of muscarinic receptor autoantibodies on parasympathetic neurotransmission in Sjogren's syndrome. Arthritis Rheum 43:1647–1654.

Wilson AG, de Vries N, Pociot F, de Giovine FS, van der Putte LB, Duff GW. 1993. An allelic polymorphism within the human tumor necrosis factor alpha promoter region is strongly associated with HLA A1, B8, and DR3 alleles. J Exp Med 177:557–560.

